# Dandruff Is a Low‐Grade Inflammatory Condition Featuring Hyperproliferative Keratinocytes, Interleukin‐17‐Producing T Cells, and N‐Acyl Ethanolamines

**DOI:** 10.1002/iid3.70467

**Published:** 2026-06-03

**Authors:** Alexandra C. Kendall, Karishma Mohamed, Holly Linley, Marta M. Koszyczarek, Anne Chandidzura, Christopher E. M. Griffiths, Tracy Hussell, Amy E. Saunders, Anna Nicolaou

**Affiliations:** ^1^ Laboratory for Lipidomics and Lipid Biology, Division of Pharmacy and Optometry, School of Health Sciences, Faculty of Biology, Medicine and Health, Manchester Academic Health Science Centre University of Manchester Manchester UK; ^2^ Manchester Collaborative Centre for Inflammation Research, Division of Infection, Immunity & Respiratory Medicine, School of Biological Sciences, Faculty of Biology, Medicine and Health, Manchester Academic Health Science Centre The University of Manchester Manchester UK; ^3^ Dermatology Centre, Salford Royal Hospital, Manchester Academic Health Science Centre University of Manchester Manchester UK; ^4^ Lydia Becker Institute of Immunology and Inflammation, Faculty of Biology, Medicine and Health, Medical Research Centre, Manchester University NHS Foundation Trust, Manchester Academic Health Science Centre The University of Manchester Manchester UK; ^5^ NIHR Manchester Biomedical Research Centre, Manchester University NHS Foundation Trust Manchester Academic Health Science Centre Manchester UK; ^6^ Division of Biomedical and Life Sciences, Faculty of Medicine and Health Lancaster University, Bailrigg Lancaster UK

**Keywords:** endocannabinoids, IL‐17, immunity, inflammation, lipidomics, skin

## Abstract

**Background:**

Dandruff features epidermal scaling but lacks overt signs of inflammation, such as erythema. Characterisation of its inflammatory profile may improve understanding of the underlying inflammatory mechanisms and therefore indicate alternative approaches for treatment.

**Methods:**

Full‐thickness scalp biopsies were sampled from healthy volunteers and those with dandruff. Immunohistochemistry for Ki67 and keratin 16 was used to examine keratinocyte proliferation and differentiation. Mass spectrometry lipidomics was applied to profile scalp skin barrier lipids and lipid mediators. The presence of immune cells and their cytokine production was examined by flow cytometry. Cytokine and chemokine levels in scalp skin were analysed by cytometric bead array.

**Results:**

Increased keratinocyte proliferation and aberrant keratinocyte differentiation were observed in dandruff relative to healthy scalp; however, this epidermal dysregulation was not mirrored by alterations to barrier lipid levels. Decreased numbers of innate lymphoid cells and reduced expression of CD86 on antigen‐presenting cells, were the few cellular alterations observed in dandruff scalp, suggesting dandruff only subtly changes major skin immune cell populations. Despite this, increased proportions of interleukin‐17‐producing T‐cells and increased C‐C Motif Chemokine Ligand 17 (CCL17) levels were observed in dandruff scalp, indicative of a low‐grade, mixed Type‐2/Type17 inflammatory response. Increased levels of N‐acyl ethanolamines, including the endocannabinoid anandamide, as well as linoleoyl‐ and oleoyl‐ethanolamine, were also observed in dandruff scalp which could be responsible for suppressing inflammation to sub‐clinical levels.

**Conclusion:**

These findings indicate that, contrary to being a mere flaking disorder, dandruff involves an altered inflammatory environment consistent with low‐grade inflammation.

## 1 Introduction

Dandruff is a common condition affecting 10%–50% of the population, irrespective of ethnicity and with a bias towards males [1, 2]. It is characterised by an itchy, flaky scalp without overt clinical signs of inflammation [3, 4]. A central hypothesis on the aetiology of dandruff is the involvement of the lipophilic yeast *Malassezia* [5], and anti‐fungal treatments reduce scaling and itch [6]. However, *Malassezia* comprise the predominant fungal species of normal microbiota and is harboured asymptomatically in many individuals [7]. Furthermore, altered prevalence of bacteria such as *Staphylococcus epidermidis* and *Cutibacterium acnes* [8, 9, 10] suggests that host‐bacterial interactions may also contribute to dandruff.

Dandruff is considered non‐inflammatory due to a lack of clinical signs such as erythema, but symptoms such as itching and flaking indicate possible underlying immunological and physical barrier disruption. Increased ‘immune response’ transcript levels [11] and higher levels of interleukin (IL)−8 [12] and tumour necrosis factor (TNF)‐α [13] in dandruff *stratum corneum* (SC), suggest immune involvement. Conversely, increased IL‐1Ra (IL‐1 receptor antagonist)/IL‐1α ratio and reduced IL‐1α concentrations, indicate reduced IL‐1 signalling in dandruff [12, 13]. Other than analysis of SC cytokines, there is a lack of research into the immune involvement in the epidermis and dermis of dandruff scalp skin, and analysis of the immune cells would add critical understanding of this condition.

There is evidence of cutaneous lipid dysregulation in dandruff, with excessive sebum production similar to other scalp‐flaking disorders, including seborrhoeic dermatitis and cradle cap. Additionally, hormonally‐controlled sebum production peaks in adolescence, corresponding with the incidence of dandruff [14]. Although higher sebum triacylglycerol (TAG) production could create conditions favourable to the development of lipophilic yeasts (e.g., *Malassezia*), it does not provide clear causality as dandruff is prevalent in all ages. Transcriptomic analysis of full‐thickness scalp biopsies revealed downregulated expression of genes involved in lipid metabolism, including fatty acid, cholesterol, and sphingolipid synthesis [11], reflecting decreases in SC sphingoid bases, free fatty acids, TAG, and cholesteryl esters (CE) reported in other studies [12, 15].

Skin barrier defects seen in dandruff, including increased transepidermal water loss and reduced skin hydration [16], could be influenced by epidermal lipids, including the SC acylceramides, associated with barrier disruption in atopic dermatitis and psoriasis [17]. Some evidence of reduced acylceramide prevalence in dandruff SC [15] has led to the proposal that dandruff may be a mild form of one of these conditions [18]. Bioactive lipid mediators of inflammation and immunity, including the cyclooxygenase and lipoxygenase‐derived eicosanoids, and endocannabinoid‐like *N*‐acyl ethanolamines (NAE), have signalling roles and could be key drivers of dandruff [19], but currently there are few studies exploring this. Finally, dandruff is associated with hyperproliferation, parakeratosis and altered differentiation of keratinocytes [20, 21, 22], and reduced expression of keratins K1, K10, K11, and K17 in the SC [12, 23], indicative of the dysregulated keratinocyte proliferation and differentiation common in inflammatory skin disease.

We hypothesise that dandruff is a low‐grade inflammatory skin disorder associated with increased immune cell activity and lipid metabolism. To examine this, we assessed skin biopsies from healthy and dandruff‐affected scalp for immune cells, immune mediators, and lipid alterations, to determine immunological and physical skin barrier changes. The resulting cutaneous inflammatory profiles, including immune cell types, chemokine and cytokine expression, comprehensive lipid analyses, and analysis of keratinocyte differentiation and proliferation, reveal intrinsic inflammatory changes in dandruff‐affected scalp skin, which could inform future approaches to management of the condition.

## 2 Materials and Methods

### 2.1 Clinical Study Design

Ethical approval was granted by NRES Committee Northwest – Greater Manchester West (14/NW/1233). The study was performed in accordance with the Declaration of Helsinki; written informed consent was obtained before volunteer participation. Two full‐thickness skin punch biopsies (4 mm; Militex Inc, York, PA) were taken from the vertex or occipital scalp regions of healthy volunteers (*n* = 29), or dandruff‐affected volunteers (*n* = 29; see Supplementary Table 1 for volunteer demographic data) during a single visit to the Dermatology Centre, Salford Royal Hospital NHS Foundation Trust. Volunteers with dandruff received a clinical diagnosis of dandruff characterised by non‐palpable, non‐adherent fine scales without visible inflammation, affecting the scalp only. Volunteers were excluded if they had a history of other skin disease, used anti‐inflammatory or anti‐fungal agents in the previous 2 weeks, or suffered hair loss. Samples for immediate immune cell and cytokine analysis were placed in PBS at 4°C and were processed as quickly as possible, usually commencing the protocol within an hour of sampling; samples for lipid analysis were snap‐frozen and stored at −80°C. Samples were chosen for each type of analysis based the volunteer availability to provide samples in the morning (fresh tissue – immune cell and cytokine analysis) or later in the day (frozen tissue – lipid analysis), with consideration to match the volunteer demographics between the healthy and dandruff groups wherever possible. In the absence of existing data at the outset of this study, the sample size (*n* = 5–8) was selected on pragmatic grounds based on our previous work on dermatoses, and the availability of volunteer samples.

### 2.2 Fluorescent Immunohistochemistry

OCT‐embedded biopsies (Klinipath, Duiven, Netherlands) were sectioned (5 µm) and fixed in ice‐cold acetone. When using a Tyramide signal amplification kit, endogenous peroxidase activity was quenched with 2% H_2_O_2_ (15 min; room temperature). Sections were blocked with 1% BSA then incubated with primary then secondary antibodies for at least 1 h. Antibodies used: rabbit anti‐Ki67 [ab15580], rabbit anti‐Keratin 16 [ab53117] (AbCam, Cambridge, UK), anti‐rabbit AlexaFluor488 secondary antibody (to detect keratin 16; Invitrogen), HRP‐conjugated anti‐rabbit secondary antibody, using a Tyramide signal amplification kit (to detect Ki67; Invitrogen). Sections were mounted with Prolong Gold antifade reagent with DAPI (Invitrogen) and images acquired using a 20× Plan Apo objective using the 3D Histech Pannoramic 250 Flash II slide scanner and viewed using Case Viewer software (3DHistech, Budapest, Hungary). Epidermal keratin 16 staining intensity was quantified using ImageJ. Ki67 staining was quantified by dividing the number of positively‐stained basal keratinocytes by the basal layer length calculated using ImageJ.

### 2.3 Flow Cytometry to Analyse Immune Cell Populations

Biopsies were split into epidermis and dermis (Dispase II; 1 mg/mL; Roche, Basel, Switzerland), then digested in collagenase (0.5 Wunch units/mL; Roche) at 37°C for 6 h (epidermis) or 18 h (dermis). Cells were strained (70 µm), counted (Countess cell counter; Invitrogen, Carlsbad, CA), incubated on ice for 30 min with human IgG (50 µg/mL; Sigma) and live/dead fixable blue dead cell stain (Invitrogen). Cells were stained with primary antibodies (Supplementary Table 2), washed and fixed with Fix/Perm buffer (eBioscience, San Diego, CA).

For IL‐17 and IL‐22 analysis, epidermis and dermis were pooled for collagenase digestion (18 h). Cells were incubated with Brefeldin A (10 µM; 5 h) before antibody staining against extracellular markers, and fixation. Cells were incubated in permeabilisation buffer (eBioscience), stained with cytokine antibodies and analysed (Becton Dickinson (BD) Fortessa flow cytometer). Data from healthy and dandruff volunteers were analysed using FlowJo software (Tree Star, Ashland, OR).

### 2.4 Lipid Extractions and Analysis by UPLC‐MS/MS and UHPSFC‐MS

NAE and ceramides were extracted and analysed as previously described [24, 25, 26]. Briefly, dermis and epidermis were separated and homogenised in chloroform:methanol (2:1) with deuterated internal standards (20 ng AEA‐*d*8, Cayman Chemical, Ann Arbor, MI; 50 pmol CER[N(25)S(18)], Avanti Polar Lipids, Alabaster, AL). Lipid extracts were analysed by ultraperformance liquid chromatography coupled to electrospray ionisation tandem mass spectrometry (UPLC/ESI‐MS/MS).

Eicosanoids were extracted and analysed as previously described [24, 26]. Briefly, frozen biopsies were separated into dermis and epidermis, and homogenised in methanol (15%) with deuterated internal standards (20 ng each of PGB_2_‐*d*4, 12‐HETE‐*d*8, 8,9DHET‐*d*11, and 8(9)EET‐*d*11; Cayman Chemical). Lipid extracts were semi‐purified by solid‐phase extraction and analysed by UPLC/ESI‐MS/MS.

Cellular lipids, including TAG, diacylglycerols (DAG), phosphatidylcholines (PC), phosphatidylethanolamines (PE), CE, and sphingomyelins (SM) were extracted and analysed as previously described [27, 28]. Briefly, dermis and epidermis were separated, homogenised in chloroform:methanol (2:1) with deuterated internal standards (SPLASH LipidoMIX cocktail (Avanti Polar Lipids); 18:1 CE‐*d*7, cholesterol‐*d*7, 18:1‐18:1‐*d*9 SM, 15:0‐18:1‐*d*7 PC, 15:0‐18:1‐*d*7 PE, 15:0‐18:1*‐d*7‐15:0 TAG, and 15:0‐18:1‐*d*5 DAG). Lipid extracts were analysed by ultra high‐performance supercritical fluid chromatography with quadrupole time‐of‐flight mass spectrometry (UHPSFC‐MS); lipids identified and quantitated using Progenesis (NonLinear Dynamics, Newcastle, UK).

Lipid levels were normalised against tissue protein content, quantified using Bio‐Rad protein assay II (Bio‐Rad, Watford, UK).

### 2.5 Cytokine and Chemokine Analysis

Skin biopsies were incubated (37°C; 20 h) in media (RPMI containing 10% heat‐inactivated FBS, 1× penicillin and streptomycin, 2 mM L‐glutamine, 1× non‐essential amino acids, 25 µM β‐mercaptoethanol, and 20 mM HEPES). Secreted cytokines and chemokines were quantified using LegendPlex kits (Biolegend, San Diego, CA; Supplementary Table 3), and a BD FACSVerse flow cytometer. Data were analysed using LegendPlex software (VigeneTech, Carlisle, MA); values below the level of detection (determined as the standard with the lowest concentration that gave a higher reading than the negative control) were assigned a value of ‘0’.

### 2.6 Statistical Analysis

Data were analysed by Mann–Whitney tests, performed using GraphPad Prism. In all analyses, *p* < 0.05 was considered statistically significant.

## 3 Results

### 3.1 Keratinocytes in Dandruff‐Affected Scalp Are Hyperproliferative and Show Altered Differentiation

**Figure 1:**
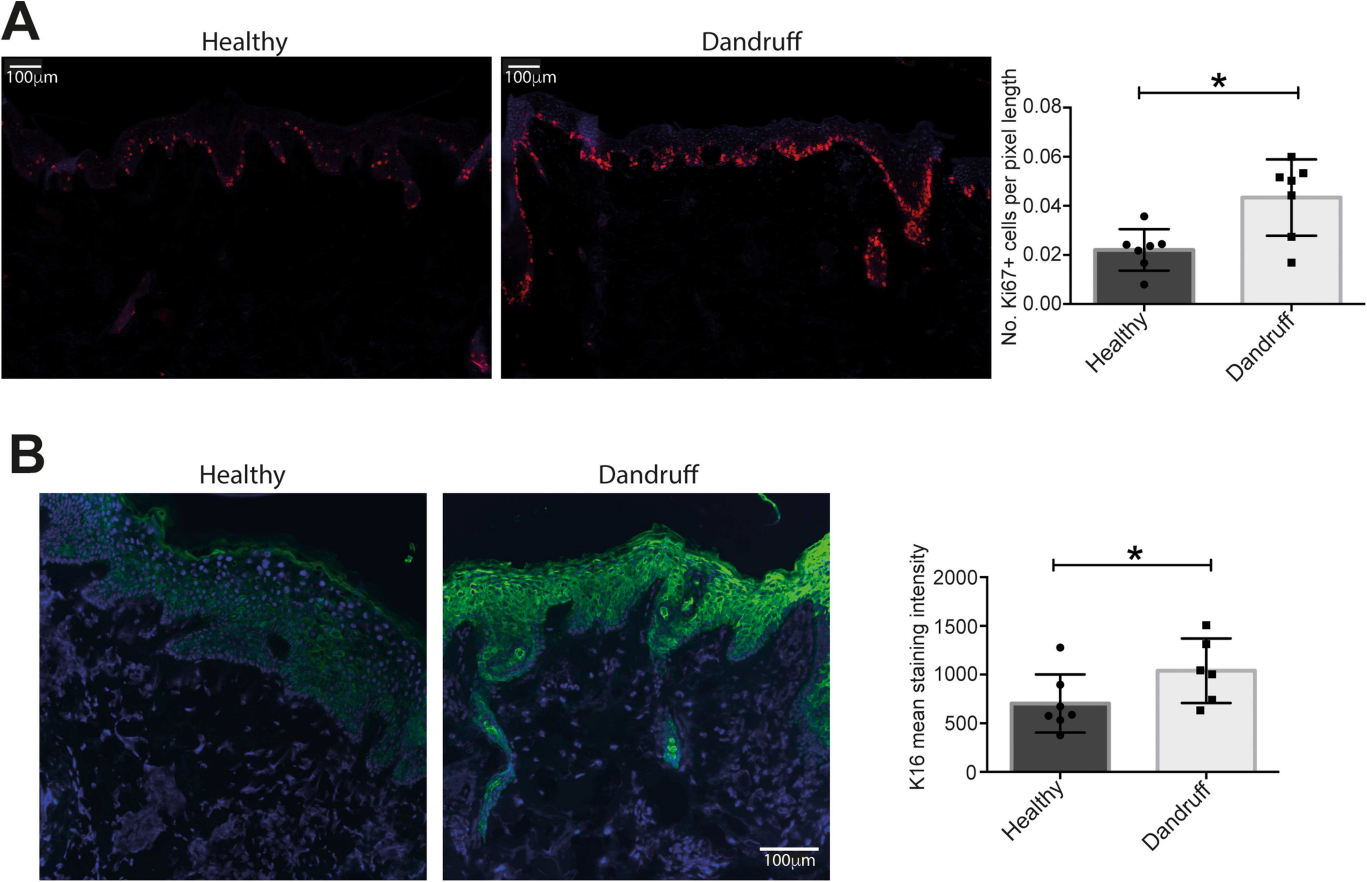
Keratinocytes in dandruff‐affected scalp are hyperproliferative and show altered differentiation. Healthy and dandruff skin scalp sections were analysed for Ki67 (A) and Keratin 16 (B). Representative images and summary data are shown. Keratin 16 staining intensity between hair follicles, and the number of Ki67‐stained cells in the basal layer, per pixel length of a line drawn through the basal layer, were quantitated using ImageJ. Scale bars represent 100 µm, *n* = 7, *p < 0.05.

Epidermal keratinocytes are critical components of the skin barrier, and inflammatory diseases with impaired barrier function commonly involve hyperproliferation and altered keratinocyte differentiation [29, 30]. Dandruff is characterised by scalp flaking and has previously been shown to involve keratinocyte hyperproliferation [21]. Therefore, we initially assessed changes in keratinocytes that may indicate the presence of inflammation in dandruff. Similar to the published data [21], we observed an increase in Ki67 staining (*p* = 0.02) by fluorescent immunohistochemistry, indicating that keratinocytes are hyperproliferative in our cohort of dandruff‐affected participants (Figure 1A). To determine whether keratinocyte differentiation is also altered in dandruff, we examined K16 expression by fluorescent immunohistochemistry. K16 is not usually expressed by healthy intrafollicular epidermis, but is increased in psoriasis where aberrant keratinocyte differentiation is observed [31]. K16 expression was observed in dandruff (*p* = 0.04) (Figure 1B), suggesting that keratinocyte differentiation is perturbed in this condition.

### 3.2 Epidermal Lipids Show Some Differences Between Healthy and Dandruff‐Affected Scalp Skin

Dandruff is associated with barrier defects, and we observed hyperproliferation and perturbed differentiation of keratinocytes in dandruff (Figure 1A,B); therefore, we examined the cellular lipids contributing to the physical integrity of the skin barrier measuring lipid classes such as TAG, DAG, PC, PE, CE, SM, and CER. Dandruff skin showed increased epidermal SM (*p* = 0.049) and decreased dermal DAG (*p* = 0.049) (Supporting Information Figure S1A), compared with healthy. Detailed assessment of epidermal long‐chain acylceramides of the CER[EOS], CER[EOH], and CER[EOP], classes revealed no significant changes in dandruff‐affected epidermis (Supporting Information Figure S1B). These data indicate that although there is aberrant keratinocyte differentiation and proliferation in dandruff, the overall lipid composition of the whole epidermis is only minimally altered.

### 3.3 Innate Lymphoid Cells Are Decreased in Dandruff‐Affected Scalp Skin

**Figure 2:**
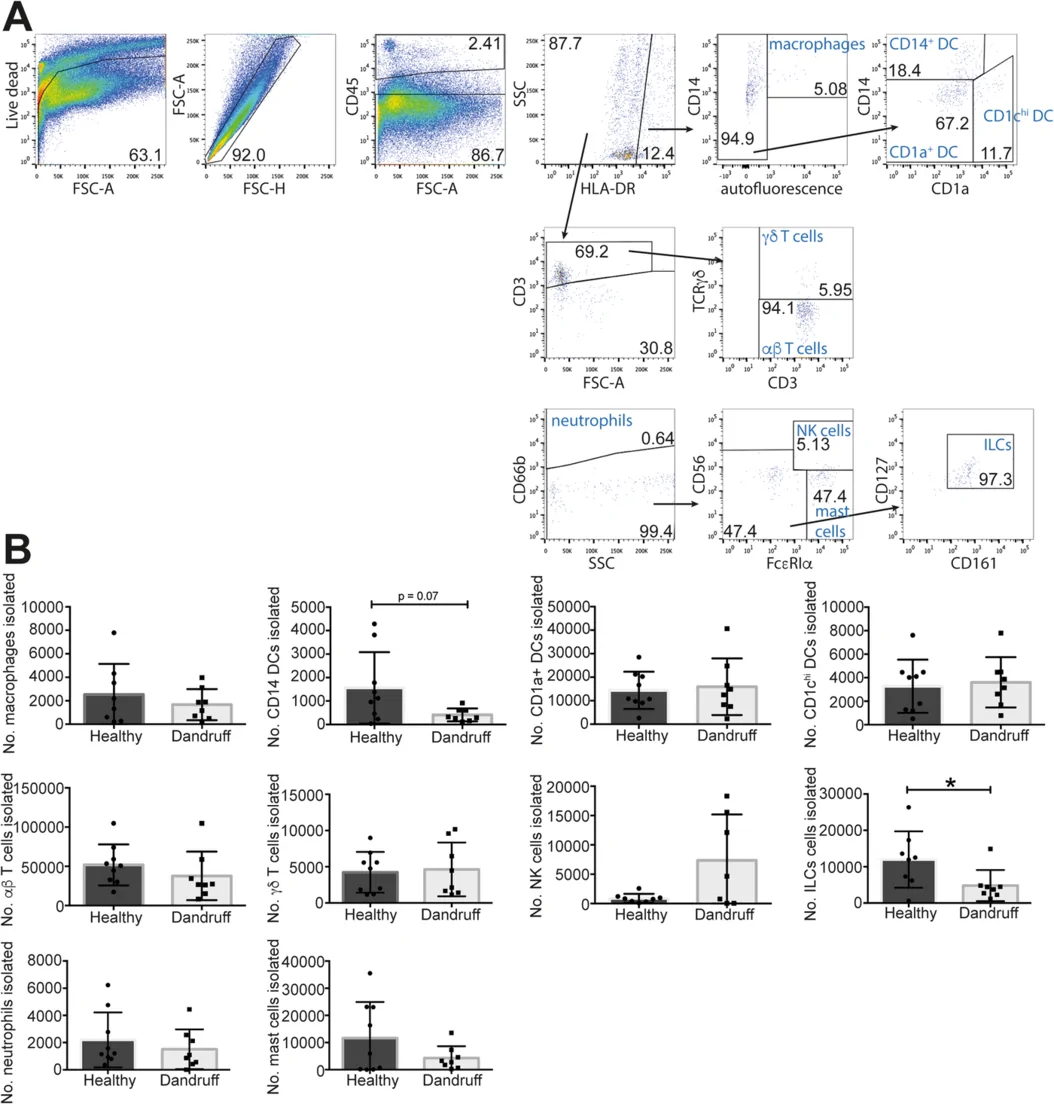
Innate lymphoid cells are reduced in dandruff scalp. Cells were isolated from scalp skin punch biopsies by enzymatic digestion, and analysed by flow cytometry. Representative flow cytometry plots show the gating strategy in dermis samples using two different staining panels (A and B). Numbers indicate the proportion of parent gate. Cell populations isolated from 4 mm full‐thickness skin biopsies are shown in panel C. *n* = 8, *p < 0.05.

Since keratinocyte hyperproliferation and dysregulated differentiation indicate epidermal barrier issues, we examined immune cell populations in healthy and dandruff‐affected full‐thickness scalp biopsies using flow cytometry (gating shown in Figure 2A). Decreased numbers of innate lymphoid cells (ILC; *p* = 0.049) were observed in dandruff skin (Figure 2B). A subset of dandruff volunteers (4 out of 8 samples analysed) had increased numbers of NK cells, and there was a suggestion of decreased CD14‐expressing dendritic cells (DC; *p* = 0.07) in dandruff, but neither was significantly different when comparing the whole dandruff cohort with healthy volunteers (Figure 2B), possibly reflecting differing degrees of inflammation in different people.

### 3.4 IL‐17‐Producing T Cells and CCL17 Levels Are Elevated in Dandruff‐Affected Scalp Skin

**Figure 3:**
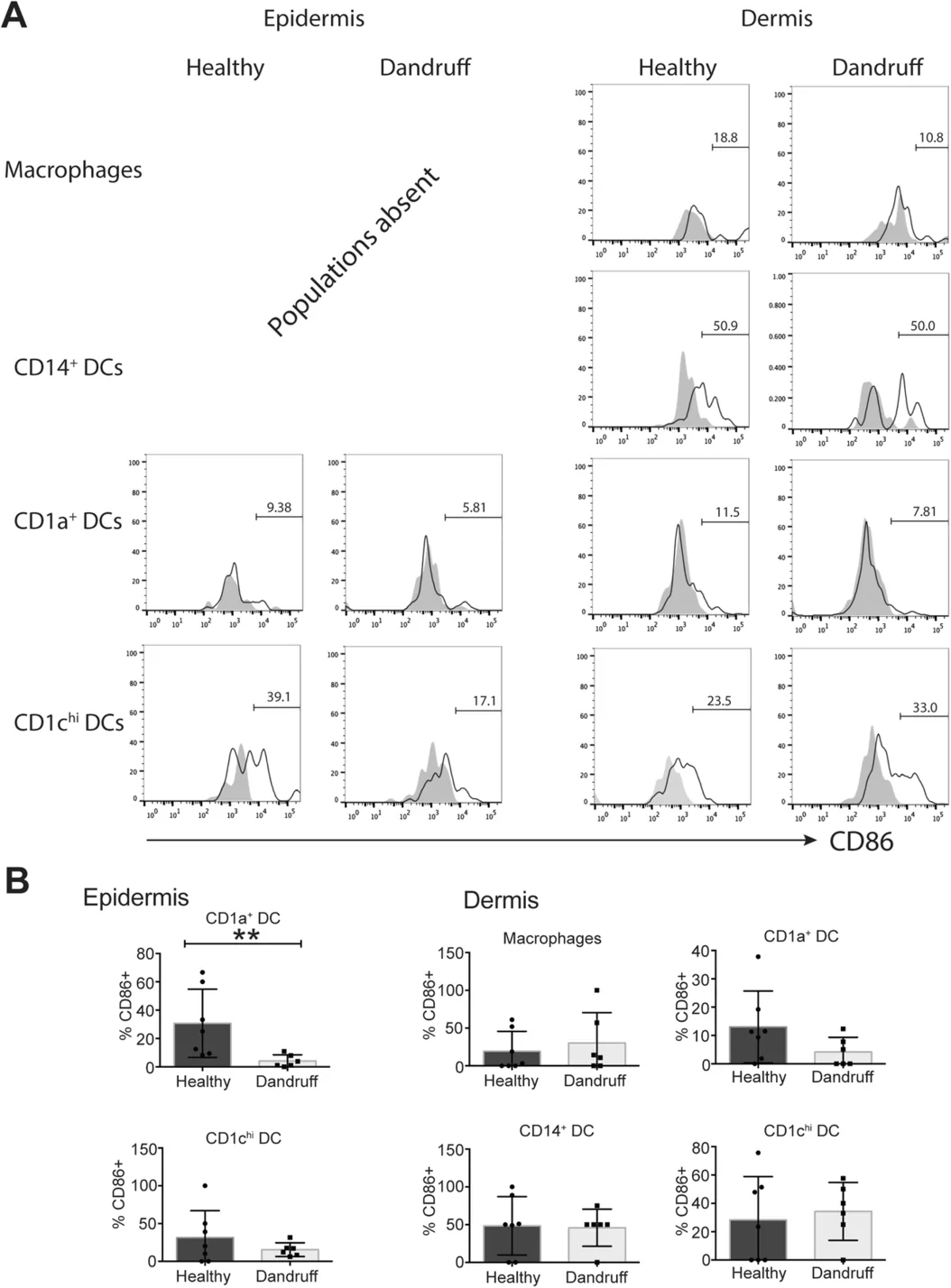
Epidermal CD1a^+^ dendritic cells express lower levels of CD86 in dandruff. Cells were isolated from scalp skin punch biopsies by enzymatic digestion, and expression of the co‐stimulatory molecule CD86 on antigen‐presenting cell populations was analysed by flow cytometry (representative flow cytometry plots for each cell population shown in panel A). The proportion of CD86‐positive cells differed in the epidermis but not the dermis (B). *n* = 7 (healthy) or 6 (dandruff), **p < 0.01.

Although overall immune cell population numbers were similar in dandruff‐affected and healthy skin, the immune cells may differ in their level of biological activity. Therefore, antigen‐presenting cell (APC) activation was examined by measuring co‐stimulatory molecule expression. Given the association between *Malassezia* and dandruff [4, 5, 32, 33], we hypothesised that APCs may be activated in dandruff‐affected scalp with increased expression of co‐stimulatory molecules such as CD80 and CD86. CD80 was not detected in scalp skin (data not shown), and surprisingly, CD86 expression was reduced in epidermal CD1a^+^ DC in dandruff‐affected scalp, suggesting that skin APCs are less activated or have a tolerogenic phenotype in dandruff (Figure 3A,B).

**Figure 4:**
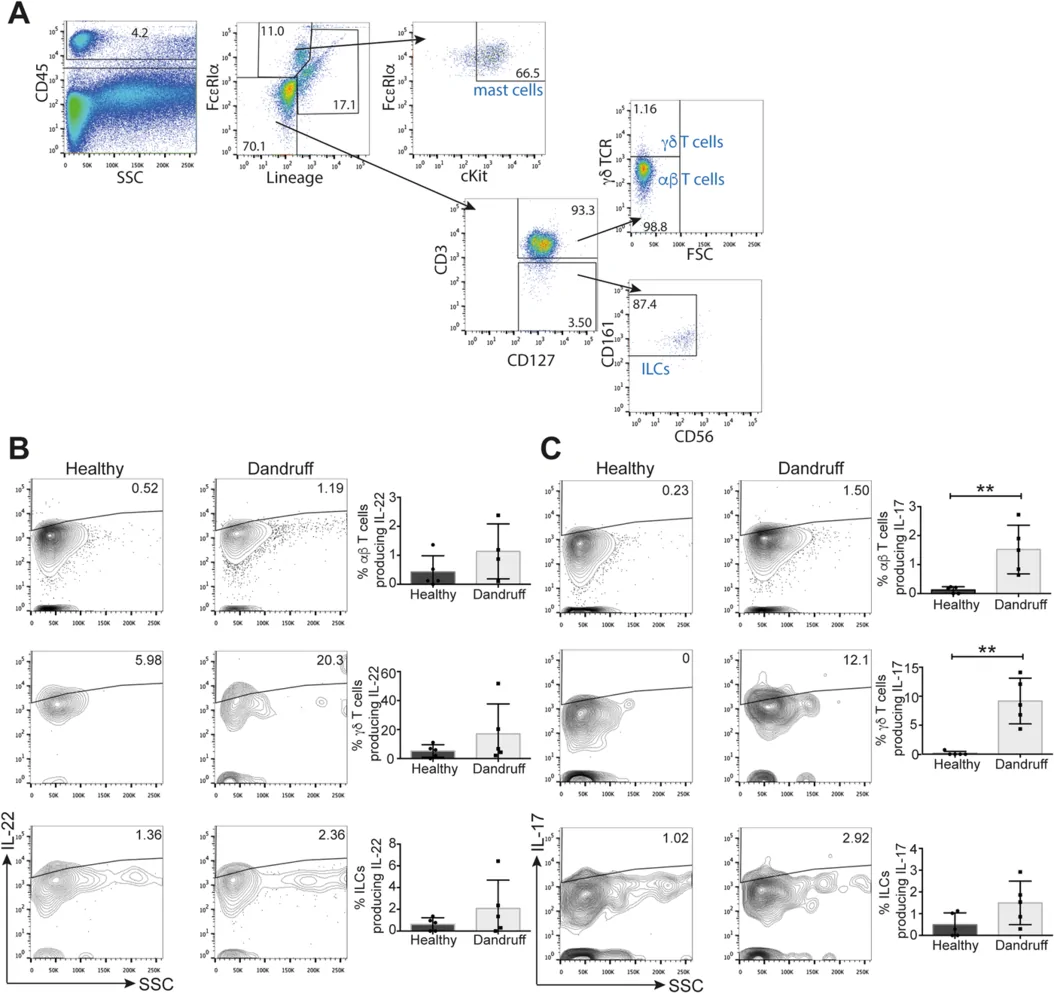
Dandruff scalp skin has increased numbers of IL‐17‐producing T cells. Cells were isolated from full‐thickness scalp skin punch biopsies by enzymatic digestion, and analysed by flow cytometry (representative flow cytometry plots to show the gating strategy for each cell population shown in panel A). Representative flow cytometry plots show IL‐22 production (B) and IL‐17 production (C) in each cell population. *n* = 5, **p < 0.01.

To further explore immune cell activity in dandruff we examined cytokine production in full‐thickness scalp skin using flow cytometry. IL‐17 contributes to changes in keratinocyte differentiation, and IL‐17 and IL‐22 are critical drivers of inflammatory skin diseases, including psoriasis [34, 35] and are key to combating fungal infections [36, 37], such as *Malassezia*. Therefore, IL‐22 and IL‐17 production were measured by intracellular flow cytometry. Increased proportions of IL‐17‐producing γδ and αβ T cells were observed in dandruff (*p* = 0.008), suggesting a potential contribution to inflammation (Figure 4C). Proportions of IL‐22‐expressing ILC and T‐cells were similar in healthy and dandruff groups (Figure 4A,B).

**Figure 5:**
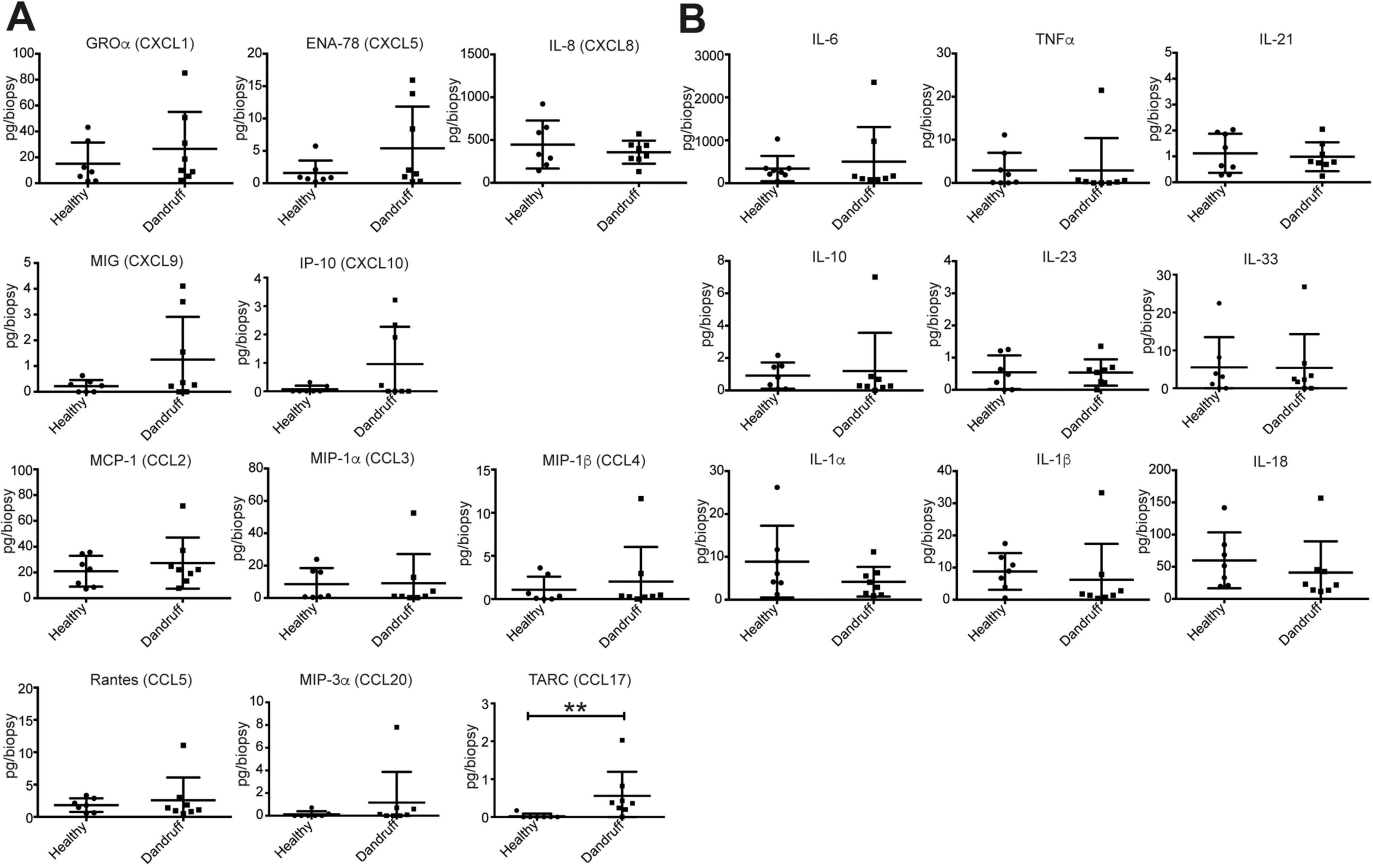
Chemokine CCL17 is elevated in dandruff‐affected scalp skin. Scalp punch biopsies (4 mm) were placed in a small volume of media overnight. Chemokines (A) and cytokines (B) released into the biopsy supernatant were measured using cytometric bead arrays (*Human Proinflammatory Chemokine, Human Th Cytokine*, and *Human Cytokine Panel 2* LegendPlex kits) and the data were expressed as the amount (pg) of each chemokine or cytokine present in the supernatant. *n* = 7 (healthy) or 8 (dandruff), **p < 0.01.

To determine wider ranging differences in cytokine and chemokine levels in dandruff, mediators were quantified by cytometric bead array in biopsy culture supernatants. Surprisingly, CCL17, a chemokine associated with type‐2 immune responses [38, 39, 40], was found elevated in dandruff scalp (*p* = 0.002) (Figure 5A). Although IL‐1α, −1β, −6, −10, −18, −21, −23, −33, and TNF‐α were detected in biopsy culture media samples, no differences were seen between healthy and dandruff‐affected scalp skin, and IL‐17A and F were undetectable by this method (Figure 5B).

### 3.5 N‐Acyl Ethanolamines Are Elevated in Dandruff‐Affected Dermis and Epidermis

As lipid mediators are involved in cutaneous inflammation [24], we examined their prevalence in the epidermis and dermis of full‐thickness skin biopsies, using targeted mediator lipidomics.

**Figure 6:**
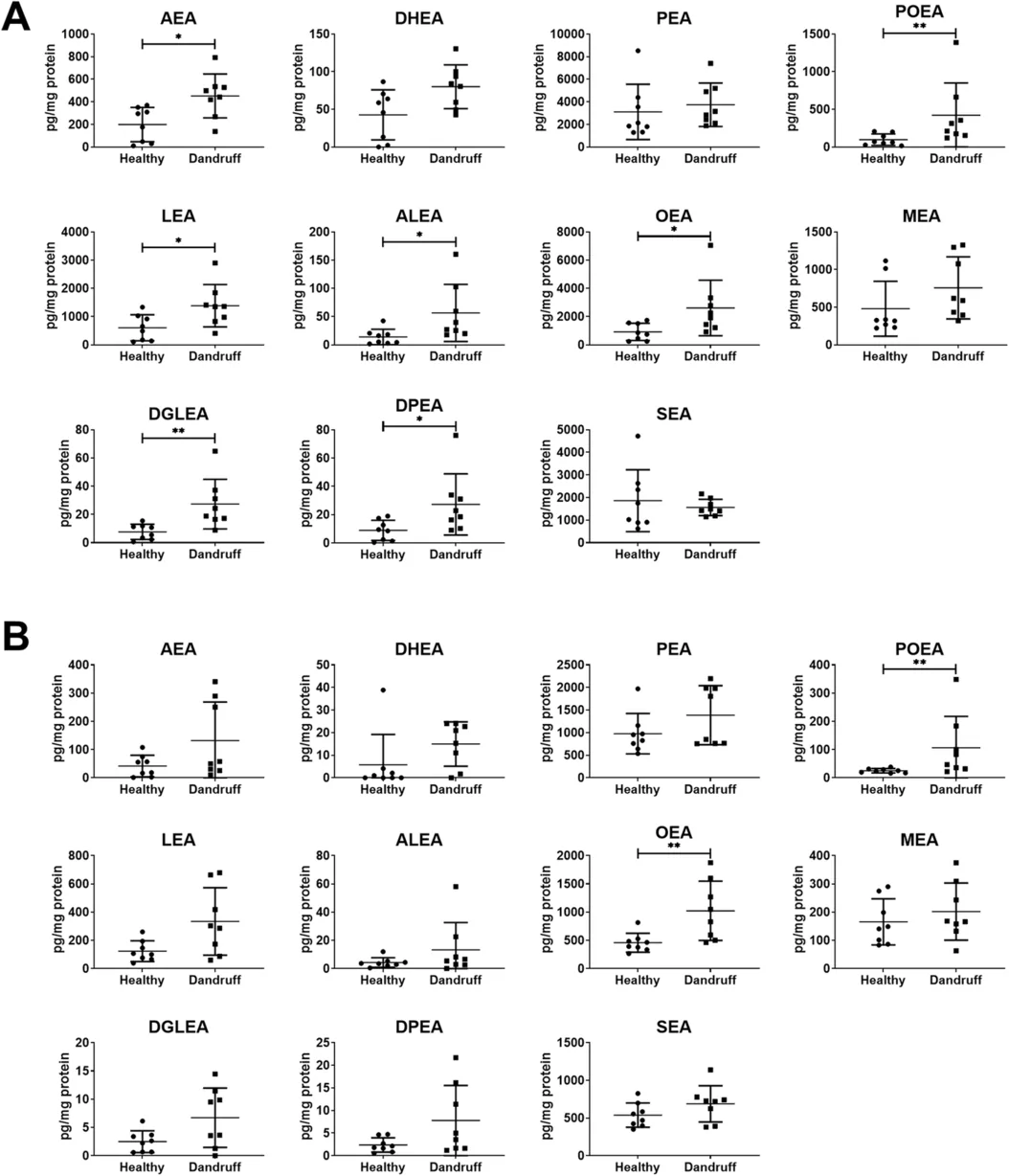
Dandruff‐affected scalp skin expresses increased levels of *N*‐acyl ethanolamines in the epidermis (A) and dermis (B). Endocannabinoids and related NAE were measured in dermis and epidermis using UPLC/ESI‐MS/MS. Data are expressed as mean ± SD, *n* = 8, *p < 0.05, **p < 0.01.

Eicosanoids, octadecanoid, and docosanoid species are potent mediators of inflammation with chemotactic properties [41]. We quantitated 40 species and found no differences between healthy and dandruff dermis or epidermis (key chemoattractant eicosanoids are shown in Supporting Information Figure S2). However, dandruff epidermis revealed increased expression of several NAEs, a class of anti‐inflammatory lipid mediators also involved in irritant dermatitis [24]. Of the 15 NAE species measured, the endocannabinoid anandamide (AEA; *p* = 0.02), as well as linoleoyl ethanolamine (LEA; *p* = 0.02), α‐linolenoyl ethanolamine (ALEA; *p* = 0.01), oleoyl ethanolamine (OEA; *p* = 0.01), dihomo‐γ‐linolenoyl ethanolamine (DGLEA; *p* = 0.002), docosapentaenoyl ethanolamine (DPEA; *p* = 0.02), and palmitoleoyl ethanolamine (POEA; *p* = 0.007) were increased in dandruff‐affected epidermis (Figure 6A). OEA (*p* = 0.007) and POEA (*p* = 0.007) were found increased in the dermis (Figure 6B).

## 4 Discussion

The involvement of inflammatory mechanisms in driving dandruff has previously been unclear. Here, using full‐thickness human scalp biopsies, increased levels of several protein and lipid mediators were revealed in dandruff‐affected scalp skin, indicating underlying inflammation despite the absence of overt erythema. IL‐17, a critical driver of skin inflammation, is shown here to be produced by increased proportions of αβ and γδ T cells in dandruff, alongside elevated levels of the type‐2 inflammatory chemokine, CCL17, and the anti‐inflammatory lipids AEA, LEA, POEA, ALEA, OEA, DGLEA, and DPEA. Enhanced keratinocyte proliferation and altered keratinocyte differentiation were also shown in dandruff. Taken together these scalp skin changes likely contribute to low‐grade inflammation manifesting as dandruff.

Very few changes to skin immune cell numbers were observed with only ILCs being reduced in dandruff scalp. In the inflammatory skin diseases psoriasis and atopic dermatitis, ILCs are more abundant in diseased skin; however, there is a larger impact on the abundance of specific subsets of these cells [42, 43, 44, 45]; which should also be investigated in dandruff. It is unclear what is causing the reduced ILCs in dandruff. These cells require cytokines such as IL‐7 or IL‐15 for their maintenance [46, 47] so, the reduction in ILCs in dandruff may be due to cytokine differences. However, IL‐15 was undetectable in scalp skin, and IL‐7 was not analysed here, so the factors driving this reduction in ILCs remain unknown. ILC can restrict sebum production [48] which is relevant to dandruff as sebum changes are also associated with this condition [14], thus ILC subsets in dandruff and their activity, especially relating to sebum production, should be examined.

CD1a+ dermal DCs are a resident steady‐state population capable of Th17 and Th2 polarisation [49]. The observed reduction in CD86 expression on these cells in dandruff may reflect a lack of activation, or changes in scalp microbial communities, as CD86 expression is affected by cytokines and microbial products [50]. In contrast to the reduced CD86 expression in dandruff, in the highly inflamed state in psoriasis, CD86 is increased [51] suggesting that DCs may be less capable of T‐cell activation in dandruff. Therefore, taken alone, the immune cell population changes in dandruff are not suggestive of inflammation.

Conversely, the observed increased proportions of IL‐17 producing T cells, suggest a causative role for this cytokine in dandruff, which is supported by a genetic link between IL‐23R (upstream of IL‐17) and dandruff frequency [52]. IL‐17 plays a crucial role in psoriasis [34], and is implicated in atopic dermatitis [53] and seborrhoeic dermatitis [54] demonstrating its crucial role in skin inflammation. IL‐17 drives keratinocyte hyperproliferation and production of cytokines, chemokines, antimicrobial peptides, and matrix metalloproteinases, which can drive neutrophil recruitment, inflammation, and host defence [55, 56, 57, 58]. Anti‐fungal immunity is particularly dependent on IL‐17 [36, 37]. As increased *Malassezia* abundance has been demonstrated in dandruff [5] the increased IL‐17 production by T cells in dandruff may be in response to fungal outgrowth. IL‐17 is also important for defence against bacterial infections of the skin, such as *Staphylococcus aureus* [59, 60]. In psoriasis, changes in the skin and gut microbiome have also been demonstrated [8, 9, 10, 61, 62] which may drive inflammation, particularly via inducing IL‐17, although further clinical studies are required to determine causation vs. correlation. Dandruff is also associated with an altered abundance of bacterial communities, which correlate with severity [63] Therefore, bacterial microbiota changes in dandruff may drive the increased IL‐17 production observed, and future studies should explore causality of the increased IL‐17 production (fungal, bacterial, or other), as well as the specificity of the IL‐17‐producing αβ and γδ T cells in dandruff.

Dandruff is considered a mild form of seborrhoeic dermatitis, an inflammatory condition associated with increases in cytokines [64, 65] possibly involving IL‐17‐producing γδ T cells [54]. Although extensive cytokine changes in tissue supernatants were not observed in dandruff, increased CCL17 (type‐2 inflammatory mediator) [38, 39, 40] levels and IL‐17 production by T‐cells, reminiscent of type‐2 and −17 immunity in seborrhoeic dermatitis, were observed. It was previously shown that IL‐1α levels are reduced (or IL‐1RA:IL‐1α is increased) in dandruff epidermis [12, 13], but here similar IL‐1α levels were detected in dandruff‐affected and healthy scalp. The difference is likely due to the sample type as, in the present study, full‐thickness skin biopsies were examined and released cytokines were measured, whilst previous studies used epidermal tape strips, sampling only the SC [12, 13]. Therefore, examining the production of cytokines and chemokines by dandruff‐affected scalp, showed an increase in IL‐17 production and CCL17 levels, suggesting that a mixed type 2/17 inflammatory response is occurring. Whilst an increase in IL‐17 production, and keratinocyte hyperproliferation and altered differentiation are reminiscent of psoriasis, dandruff does not involve overt erythema, demonstrating that the inflammation in dandruff is low‐grade.

Lipid mediators can regulate the immune and inflammatory environment in skin [41], and lipid dysregulation has previously been implicated in dandruff [6, 15]. Eicosanoids and related species with inflammatory and chemotactic properties were unaltered, commensurate with the absence of severe inflammation. However, a significant increase in the concentration of NAE was observed in dandruff‐affected scalp skin. These lipid mediators are elevated in irritant dermatitis [24], although their levels in other skin‐flaking disorders remain unexplored. NAE demonstrate anti‐inflammatory properties when applied exogenously to skin, including analgesic, anti‐pruritic, and immunosuppressive qualities [66, 67, 68]. The increased levels of NAE observed here could therefore help prevent inflammation from reaching the levels seen in other skin flaking disorders. Furthermore, NAE can regulate aspects of immune cell function, and most immune cells express CB1, CB2, TRPV1, GPR55, and PPARα/γ receptors [69]; therefore the increased NAE in dandruff could contribute to changes in immune status. NAE species, including LEA and OEA, which were found upregulated in dandruff, can suppress cyclooxygenase activity [70, 71, 72], potentially explaining why eicosanoid levels were not elevated, despite other signs of inflammation. Interestingly, NAE are also known to promote sebocyte lipid production [73], which is elevated in dandruff skin [6] and may lead to positive feedback, further propagating dandruff reactions.

Further analysis of epidermal lipids indicated some lipid dysregulation, with dandruff‐affected epidermis having more SM, and dermis having less DAG compared with healthy scalp. These changes were observed in lipids extracted from whole epidermis or dermis, so they cannot be linked directly to the SC barrier, where previous work on dandruff revealed changes to ceramides and free fatty acids [15], which are of particular significance in the epidermal barrier. Given the dryness and flaking of dandruff‐affected skin, further work examining tape strip SC samples would be useful to assess lipid composition and barrier dysfunction in dandruff.

Increases in epidermal basal cell turnover and K16 expression in dandruff were seen here, which are indicative of disrupted keratinocyte differentiation, concurring with the parakeratosis observed in previous studies [22]. Enhanced keratinocyte turnover and altered differentiation are common features of skin inflammation [29], including psoriasis plaques [74] and seborrhoeic dermatitis [65], further indicating similarities between dandruff and inflammatory dermatoses.

A limitation of this work arises from the unequal sex balance of volunteer groups, in the overall cohort and within some experimental subsets, including immunohistochemistry (Figure 1), immune cell flow cytometry (Figures 2 and 3) and chemokine analysis (Figure 5). This imbalance may have affected our ability to detect sex‐related changes, although there were not any obvious differences between males and females for any of the parameters measured here. Furthermore, the age of volunteers was wide ranging to enable sufficient recruitment; however, within each experimental subset, the median age and age range of volunteers were kept as comparable as practically possible.

Another consideration arises from measuring cytokines and chemokines in the supernatant of ex vivo scalp biopsy cultures, as differential cell composition, variable cell viability, and stress responses may have affected mediator profiles. To address this, time‐from‐biopsy to culture, handling, and culture conditions were standardised across participants. Although biopsies were always taken from the vertex or occipital regions of the scalp, matching exact scalp locations between dandruff and healthy volunteers was not always possible, an additional factor which may have introduced data variability.

Overall, our data show a mixture of increased inflammatory processes, including cytokines and chemokines (IL‐17 and CCL17), as well as epidermal hyperproliferation and dysregulated differentiation, suggestive of inflammation being present in dandruff. Additionally, the observed decreased ILCs and co‐stimulatory molecule expression on DCs, and upregulation of anti‐inflammatory NAE lipids, suggested increased activity of mechanisms inhibiting inflammation, potentially responsible for limiting the magnitude of inflammation observed in dandruff. Further work should determine which factors are causative, how the interplay between bioactive lipids, chemokines, and immune cells contributes to dandruff, and confirm links between dandruff and other skin disorders.

## Author Contributions

**Alexandra C. Kendall:** data curation, formal analysis, investigation, methodology, supervision, visualisation, writing – original draft. **Karishma Mohamed:** investigation. **Holly Linley:** investigation. **Marta M. Koszyczarek:** investigation. **Anne Chandidzura:** investigation. **Christopher E. M. Griffiths:** conceptualisation, funding acquisition, writing – review and editing. **Tracy Hussell:** conceptualisation, funding acquisition. **Amy E. Saunders:** conceptualisation, data curation, formal analysis, methodology, supervision, visualisation, writing – original draft. **Anna Nicolaou:** conceptualisation, project administration, funding acquisition, methodology, writing – original draft, supervision.

## Ethics Statement

Ethical approval was granted by NRES Committee Northwest – Greater Manchester West (14/NW/1233).

## Consent

The study was performed in accordance with the Declaration of Helsinki; written informed consent was obtained before volunteer participation.

## Conflicts of Interest

Alexandra C. Kendall is named on a patent with Unilever R&D, UK. Karishma Mohamed, Holly Linley, Marta M. Koszyczarek, Anne Chandidzura, and Tracy Hussell have no conflicts of interest to declare. Christopher E. M. Griffiths received grant funding from Unilever R&D, UK, received grants from Almirall, Amgen, and Janssen, received consulting fees from BMS, GSK, Boehringer Ingelheim, and Janssen, received payment or honoraria from BMS, Lilli, Janssen, and Novartis, participates on a data safety monitoring board or advisory board for BMS, and has stock in CGSkincare. Amy E. Saunders was supported by a Wellcome Trust Sir Henry Dale Fellowship and received grant funding from Unilever R&D, UK. Anna Nicolaou received grant funding from Unilever R&D, UK, is named on a patent with Unilever R&D, UK, and has roles as ISSFAL executive board member, EuroFedLipid Lipidomics Division chair, and member of the BBSRC Pool of Experts.

## Supporting information


**Figure S1:** Healthy and dandruff‐affected scalp skin show similar levels of cellular lipid species (A) and epidermal acylceramides (B).Lipids were extracted from healthy and dandruff epidermis and dermis and analysed by UHPSFC/ESI‐QToF while epidermal ceramides were analysed by UPLC/ESI‐MS/MS. Data are expressed as mean ± SD, n = 8.

**Figure S2:** Eicosanoid expression does not differ between healthy and dandruff‐affected scalp skin.Eicosanoids and related docosanoids and octadecanoids were measured in dermis (A) and epidermis (B) using UPLC/ESI‐MS/MS. There were no significant differences between healthy and dandruff skin. Error bars represent mean ± SD, n = 8.

**Table S1**: Volunteer demographic data. **Table S2**: Antibodies used for flow cytometry. **Table S3:** Analytes measured in biopsy supernatant.

## Data Availability

The data that support the findings of this study are available from the corresponding author upon reasonable request.
