# Supplementary material for: Dandruff Is a Low‐Grade Inflammatory Condition Featuring Hyperproliferative Keratinocytes, Interleukin‐17‐Producing T Cells, and N‐Acyl Ethanolamines
Source: Immun Inflamm Dis. 2026 Jun 3;14(6):e70467. doi: 10.1002/iid3.70467 (PMC13581083; doi:10.1002/iid3.70467)
Supplement: Supplementary file 1 [file IID3-14-e70467-s001.docx]

Supplementary Table 1: Volunteer demographic data

| **Experimental Groups** | **Number of samples** | | **Male, Female** | | **Ages yrs (range) median** | |
| --- | --- | --- | --- | --- | --- | --- |
|  | **Healthy** | **Dandruff** | **Healthy** | **Dandruff** | **Healthy** | **Dandruff** |
| **All participants** | 29 | 29 | 8M, 21F | 17M, 12F | (19-62) 43 | (18-73) 37 |
| **Immunohistochemistry** | 7 | 7 | 0M, 7F | 4M, 3F | (30-59) 44 | (26-73) 50 |
| **Immune cell numbers by flow cytometry** | 8 | 8 | 1M, 7F | 6M, 2F | (25-47) 32 | (19-61) 41 |
| **CD86 by flow cytometry** | 7 | 6 | 1M, 6F | 4M, 2F | (25-58) 32 | (21-61) 42.5 |
| **Cytokines by flow cytometry** | 5 | 5 | 3M, 2F | 2M, 3F | (19-52) 31 | (32-67) 36 |
| **Chemokine and cytokines (bead array)** | 7 | 8 | 0M, 7F | 4M, 4F | (25-59) 44 | (26-73) 48.5 |
| **Lipidomics** | 8 | 8 | 4M, 4F | 5M, 3F | (25-62) 50 | (24-57) 34 |

Supplementary Table 2: Antibodies for flow cytometry

|  | **Specificity** | **Conjugate** | **Clone** | **Manufacturer** |
| --- | --- | --- | --- | --- |
| **Panel 1*** | CD1a | PerCPeF710 | HI149 | eBioscience |
|  | CD19 | APCCy7 | SJ25C1 | BD Bioscience |
|  | HLA-DR | eF450 | LN3 | eBioscience |
|  | CD80 | BV510 | L307.4 | BD Bioscience |
|  | CD86 | BV650 | 2331 | BD Bioscience |
|  | CD45 | BV786 | HI30 | BD Bioscience |
|  | CD14 | PECy7 | 61D3 | eBioscience |
|  | CD123 | PECF594 | 7G3 | BD Bioscience |
|  |  |  |  |  |
| **Panel 2** | HLA-DR | FITC | G46-6 | BD Bioscience |
|  | FcεRIα | PerCPeF710 | AER-37 | eBioscience |
|  | CD3 | APCCy7 | SK7 | BD Bioscience |
|  | CD66b | V450 | G10F5 | BD Bioscience |
|  | CD127 | BV510 | HIL-7R-M21 | BD Bioscience |
|  | CD117 | BV650 | 104D2 | BD Bioscience |
|  | CD161 | BV711 | DX12 | BD Bioscience |
|  | CD45 | BV786 | HI30 | BD Bioscience |
|  | TCRγδ | PECF594 | B1 | BD Bioscience |
|  | CD56 | PECy7 | CMSSB | eBioscience |
|  |  |  |  |  |
| **Panel 3 extracellular** | CD11c | FITC | 3.9 | eBioscience |
|  | CD14 | FITC | 61D3 | eBioscience |
|  | CD19 | FITC | HIB19 | eBioscience |
|  | CD66b | FITC | G10F5 | eBioscience |
|  | CD123 | FITC | 6H6 | eBioscience |
|  | FcεRIα | PerCPeF710 | AER-37 | eBioscience |
|  | CD117 | APC-R700 | YB5.B8 | BD Bioscience |
|  | CD3 | APCCy7 | SK7 | BD Bioscience |
|  | CD127 | BV510 | HIL-7R-M21 | BD Bioscience |
|  | CD56 | BV605 | NCAM16.2 | BD Bioscience |
|  | TCRγδ | BV650 | B1 | BD Bioscience |
|  | CD161 | BV711 | DX12 | BD Bioscience |
|  | CD45 | BV786 | HI30 | BD Bioscience |
|  | NKp44 | PE | P44-8 | BD Bioscience |
|  | IL-21R | PECF594 | 17A12 | BD Bioscience |
| **intracellular** | IL-17 | eF660 | SHLR17 | eBioscience |
|  | IL-21 | BV421 | 3A3-N2.1 | BD Bioscience |
|  | IL-22 | PECy7 | 22URTI | eBioscience |

* The FITC channel was also recorded for panel 1 as a measure of autofluorescence.

Supplementary Table 3: Cytokines and chemokines analysed by bead array

| **Kit used (LegendPlex, Biolegend)** | **Analytes measured** |
| --- | --- |
| human Th cytokine | IL-2, IL-4, IL-5, IL-6, IL-9, IL-10, IL-13, IL-17A, IL-17F, IL-21, IL-22, IFN-γ and TNF-α |
| human cytokine panel 2 | TSLP, IL-1α, IL-1β, GM-CSF, IFN-α2, IL-23, IL-12p40, IL-12p70, IL-15, IL-18, IL-11, IL-27, and IL-33. |
| human proinflammatory chemokine | MCP-1 (CCL2), RANTES (CCL5), IP-10 (CXCL10), Eotaxin (CCL11), TARC (CCL17), MIP-1α (CCL3), MIP-1β (CCL4), MIG (CXCL9), MIP-3α (CCL20), ENA-78 (CXCL5), GROα (CXCL1), I-TAC (CXCL11) and IL-8 (CXCL8). |
